# Dry-Cured Meat Products According to the Smoking Regime: Process Optimization to Control Polycyclic Aromatic Hydrocarbons

**DOI:** 10.3390/foods9010091

**Published:** 2020-01-15

**Authors:** Maria João Fraqueza, Marta Laranjo, Susana Alves, Maria Helena Fernandes, Ana Cristina Agulheiro-Santos, Maria José Fernandes, Maria Eduarda Potes, Miguel Elias

**Affiliations:** 1 CIISA-Centro de Investigação Interdisciplinar em Sanidade Animal, Faculdade de Medicina Veterinária, Universidade de Lisboa, Avenida da Universidade Técnica, 1300-477 Lisboa, Portugal; mjoaofraqueza@fmv.ulisboa.pt (M.J.F.); susanaalves@fmv.ulisboa.pt (S.A.); helenafernandes@fmv.ulisboa.pt (M.H.F.); mjfernandes@fmv.ulisboa.pt (M.J.F.); 2 MED-Mediterranean Institute for Agriculture, Environment and Development, IIFA-Instituto de Investigação e Formação Avançada, Universidade de Évora, Pólo da Mitra, Ap. 94, 7006-554 Évora, Portugal; mlaranjo@uevora.pt (M.L.); acsantos@uevora.pt (A.C.A.-S.);; 3 Departamento de Fitotecnia, Escola de Ciências e Tecnologia, Universidade de Évora, Pólo da Mitra, Ap. 94, 7006-554 Évora, Portugal; 4 Departamento de Medicina Veterinária, Escola de Ciências e Tecnologia, Universidade de Évora, Pólo da Mitra, Ap. 94, 7006-554 Évora, Portugal

**Keywords:** cured meat products, smoking, chemical hazards, polycyclic aromatic hydrocarbons (PAHs), food safety, food quality

## Abstract

The manufacturing of dry-cured meat products usually includes a smoking step. Polycyclic aromatic hydrocarbons (PAHs) are potentially carcinogenic chemical compounds that may result from smoking. The aim of the present study was to optimize the smoking regime of traditional dry-cured meat products in order to minimize the presence of PAHs. Dry-cured sausages were submitted to different smoking regimes: (A) no smoking; (B) 20 h effective smoking; (C) 60 h effective smoking; (D) effective smoking until reaching 38%–40% weight losses. Three independent batches were produced per smoking regime, and three samples per batch were analyzed. Microbiological, physicochemical, and sensory analyses were performed. The total PAHs content was generally low and did not differ significantly in meat products submitted to the four different smoking regimes. The PAH4 and benzo(α)pyrene levels were below the established legal limits in all analyzed dry-cured sausages. Nevertheless, non-smoked sausages always showed lower PAHs values for all PAHs groups.

## 1. Introduction

Dry-cured meat products result from ancestral know-how passed onto the new generations. The meat is salted and mixed with condiments and additives, particularly nitrate or nitrite. This meat batter is afterwards fermented, dried and smoked according to traditional processes. The main objectives of this technology are to give the meat a different appearance and distinct flavors and textures and to extend its shelf-life. These meat products are considered to comply with the ‘history of safe use’ concept of European Food Safety Authority (EFSA)’s safety assessment guidance, due to the large evidence of safe production and consumption by a genetically diverse human population collected over the years [1].

However, in 2015, the International Agency for Research on Cancer (IARC) communicated an opinion from a working group of experts based on a systematized analysis of thousands of research articles concluding that the relationship between meat products and colorectal cancer was unquestionable [2]. Case–control and cohort studies demonstrated the association between meat products consumption and colorectal cancer. In fact, a significant dose–response relationship was specifically established with an increased risk of 17% when approximately 100 g of processed meat were consumed per day. Based on this strong evidence, the IARC working group classified processed meat as “carcinogenic to humans” (Group 1) [3].

The two main mechanisms involved in the increased risk of cancer by consumption of processed meat are related to the presence of N-nitroso compounds (NOCs) and polycyclic aromatic hydrocarbons (PAHs).

PAHs are ubiquitous environmental contaminants [4]; however, several authors have reported human exposure to PAHs to occur mainly through food [5,6,7]. Different sources of food contamination include the production of PAHs during the thermal processing of foods, such as grilling and smoking, contamination from food-packaging materials, and direct deposition of PAHs from the atmosphere [8].

PAHs can have severe harmful effects on human health. They have carcinogenic, mutagenic, and teratogenic properties. PAHs are formed throughout the smoking process and can be deposited on the surface of smoked meat products or barbecued meat [3,9]. Traditional cold and hot smoking are carried out in small manufacturing units by burning wood or wood chips. Under cold smoking, environment temperatures usually do not exceed 20 °C, while in hot smoking, temperatures are around 80 °C [10,11]. To prevent excessive accumulation of PAHs in smoked processed meats, it is recommended to avoid high pyrolysis temperatures and the generation of direct smoking. In modern smoking chambers, the smoke generator is located aside the smoking chamber. Moreover, this separation avoids fat dripping over the fire and minimizes the formation of toxic compounds [12]. It should be emphasized that the main products of wood pyrolysis are phenols, carbonyls, and organic acids, which are also responsible for the flavor, color, and antimicrobial properties of smoke [13].

The aim of this study was to submit a dry-cured meat product to different levels of smoking in order to minimize the presence of PAHs. Furthermore, we aimed to establish good smoking practices in order to avoid and control this hazard.

## 2. Materials and Methods

### 2.1. Cured Products Processing and Sampling

“Paio”, a traditional Portuguese dry-cured sausage (DCS), was manufactured in a local factory using commercial hybrid Iberian × Duroc pig breed meat.

Trimmings (80/20) were mechanically cut into cubes of 35 to 45 mm and mixed with red pepper (*Capsicum annuum* L.) paste (6% w/w), white wine (1% v/v), garlic (*Allium sativum* L.) paste (1% w/w), and powder laurel (*Laurus nobilis* L.) (0.006% w/w). Polyphosphates (0.15% w/w) were added to the commercial mixture AGLO P (MANE IBERICA S.A., Barcelona, Spain), while nitrates (0.003% w/w) and nitrites (0.003% w/w) were added in the form of the commercial additive NITROS 5/5 (Formulab, Moreira, Portugal). Since red pepper and garlic pastes have in their original composition approximately 17% of salt, supplementary addition of salt to the mixture was done to obtain a final concentration of 4% in the end products.

The meat batter was stored under controlled conditions at 5 °C for 48 h for ripening purposes. Afterwards, the batter was stuffed into pork natural casings of 50–55 mm.

From the initial batter, four experimental groups were submitted to different smoking regimes: (A) no smoking; (B) 20 h effective smoking (3 days of smoking); (C) 60 h effective smoking (8.5 days of smoking); (D) effective smoking until reaching 38%–40% weight losses.

The general curing procedure occurred in two steps. All DCS, except group (D), were dried in a cure chamber under controlled conditions at a temperature of 5 °C and relative humidity of 80%–85% until reaching 38%–40% weight losses, throughout the curing period or after a smoking step. Smoking was generated by burning oak (*Quercus ilex* L.) wood. Smoked DCS were indirectly exposed to smoke during approximately 7 h/day.

Three independent batches of “Paio” were produced in different working days. Three samples were collected for each of the smoking groups.

### 2.2. Microbiological Analyses

Microbiological analyses were performed following established procedures: mesophiles following ISO 4833-1 [14]; lactic acid bacteria (LAB) according to ISO 15214 [15], under anaerobiosis; staphylococci as described by Laranjo et al. [16]; enterococci as described by Talon et al. [17]; enterobacteria according to ISO 21528-2 [18]. All microbiological analyses were performed in triplicate, and the results were expressed in log colony-forming units (cfu)/g.

The detection of *Listeria monocytogenes* was not performed, because all studied products had water activity (a_W_) values below 0.92.

### 2.3. Physicochemical Analyses

#### 2.3.1. Determination of pH, a_W_, and Chlorides

DCS casings were removed, and pH values were measured with a Crison 507 (Crison, Barcelona, Spain) pH-meter following the procedures described in ISO 2917 [19]. Water activity (a_W_) was determined at 25 °C with a hygrometer (Hygroskop Rotronic DT, Zurich, Switzerland) equipped with a WA-40 probe. Salt content was confirmed through the determination of chlorides according to the Volhard method, as described in ISO 1841-1 [20].

#### 2.3.2. Color

Color was measured on cross sections of DCS with a Konica Minolta CR-400 colorimeter (Konica Minolta Inc., Tokyo, Japan) in five replicates per sample. The chromatic coordinates L* a* b* were determined using the CIELab System. All measurements were performed using the standard illuminant D65.

#### 2.3.3. Texture Profile Analysis

Texture profile analysis (TPA) was performed using a Stable Micro System TA-Hdi (Stable Micro Systems, Godalming, England) following the procedures described before [21,22] and adapted by Laranjo et al. [23] using a cylindrical flat-ended plunger (with an area of 1 cm^2^). The tests were carried out at room temperature (20 °C ± 1 °C). The samples were cut into 1 cm-thick slices, with a diameter of approximately 2.5 cm, which were compressed twice in two consecutive cycles of 50% compression with 5 s intervals between cycles, while the plunger was actioned at a constant speed of 1 mm s^−1^. Force–time curves were used to calculate the following parameters: hardness, adhesiveness, springiness, cohesiveness, resilience, and chewiness. Five replicates per sample were used.

### 2.4. Sensory Analysis

Sensory analysis was performed in a special room with the requirements described in ISO 8589 [24]. Sample preparation was done 30 min before each session. The samples of each batch and condition were cut into 3 mm-thick slices. Six slices codified with a three-digit number were randomly disposed in white dishes. The trained group included five men and five women (40–60 years old) selected according to ISO 8586-1 [25]. The group was asked to evaluate the products according to the following attributes, using a quantitative descriptive analysis (QDA^®^) with a scale ranging from 0 to 100 corresponding to ‘‘no perception” or ‘‘maximum perception”, respectively: color intensity, aroma intensity, flavor intensity, salt perception, hardness, fibrousness, succulence, off colors, off aromas, off flavors, overall appreciation. The evaluation of hardness was the exception, being communicated to the group that 50% of the scale would correspond to the optimum value. Each panelist tasted six sausages per session. Crackers and mineral water were provided, for the panelists to rinse their mouths between evaluations.

### 2.5. Polycyclic Aromatic Hydrocarbons Determination

Polycyclic aromatic hydrocarbons were determined by GC–MS using deuterated labelled PAHs, according to Alves et al. [26] and a procedure adapted from Mottier, Parisod, and Turesky [8]. The deuterium-labelled internal standard PAHs mix (LGC standards, Middlesex, UK) contained 16 PAHs (chemical purity >98%; isotope purity >99%): naphthalene-d8, acenaphthylene-d8, acenaphthene-d10, fluorene-d10, anthracene-d10, phenanthrene-d10, fluoranthene-d10, pyrene-d10, chrysene-d12, benzo[a]anthracene-d12, benzo[b]fluoranthene-d12, benzo[k]fluoranthene-d12, benzo[a]pyrene-d12, benzo[g,h,i]perylene-d12, indeno [1,2,3-c,d]-pyrene-d12, and dibenzo[a,h]anthracene-d14. Briefly, PAHs were saponified with 2M potassium hydroxide in ethanol/distilled water (9:1, v/v) under reflux for 5 h. Solid-phase extraction (SPE) was used for the purification of the PAHs, using two SPE column phases in a vacuum manifold (Varian, Palo Alto, CA, USA). First, the extract was applied into an aminopropyl SPE column (Supelco, Bellefonte, PA, USA) previously conditioned with cyclohexane and, afterwards, it was applied into a C18 SPE column (Supelco, Bellefonte, PA, USA). The PAHs were evaporated in a rotary evaporator to about 0.5 mL of volume and transferred into an amber GC vial. An internal standard (400 μL of a solution 20 μg/kg of the deuterated mixture containing 16 PAHs) was added before saponification. PAHs were analyzed by gas chromatography coupled to mass spectrometry using a GC–MS QP2010-Plus (Shimadzu, Kyoto, Japan) equipped with a SPB-5 chromatographic column (30 m × 0.25 mm × 0.25 μm film thickness, Supelco, Bellefonte, PA, USA). The chromatographic conditions were as follow: injector temperature, 250 °C; injection mode, splitless; column flow, 1.18 mL/min; carrier gas, helium; column oven temperature program, the initial temperature of 80 °C (maintained 0.5 min) was increased to 230 °C at 8 °C/min, then it was increased to 300 °C at 5 °C/min (maintained for 6 min). The mass spectrometer conditions were as follows: ion source temperature, 200 °C; interface temperature, 280 °C; ionization energy, 70 eV. The analysis was performed by selected ion monitoring (SIM). Each PAH was quantified using external calibration curves, which were constructed using commercial standard solutions containing unlabeled PAHs mixtures (Sigma-Aldrich, St. Louis, MO, USA), namely, naphthalene (NAP), acenaphthylene (ACY), acenaphthene (ACE), fluorine (FLR), phenanthrene (PHE), anthracene (ANT), fluoranthene (FLT), pyrene (PYR), chrysene (CHR), benzo[a]anthracene (BaH), benzo[b]fluoranthene (BbF), benzo[k]fluoranthene (BkF), benzo[a]pyrene (BaP), indeno[1,2,3-c,d]pyrene (IcP), dibenzo[a,h]anthracene (DhA), and benzo[g,h,i]perylene (BgP) at 13 concentrations, ranging from 0 to 30 μg/kg of sausage, and deuterated PAHs mixtures at a concentration set to 20 μg/kg. The calibration standards were injected before and after the samples, and both data sets were used to build the calibration curves.

### 2.6. Statistical Analysis

The Shapiro–Wilk test was used in order to evaluate whether the data followed a normal distribution. When not normally distributed (*p* < 0.05), some PAHs data were transformed before further analysis. For ACY, ACE, FLR, ANT, FLT, PYR, BaA, total PAHs, heavy, light, and PAH8, the data were log-transformed, whereas for NAT and PHE, the inversed and square root transformations were used, respectively. After statistical analysis, means and standard error of the means were back-transformed.

All data were analyzed with ANOVA using Statistica^TM^ v.12.0 software (1984–2014) from Statsoft (StatSoft Inc., Tulsa, OK, USA). Significant differences (*p* < 0.05) were identified using Tukey’s honest significant difference (HSD) test.

## 3. Results and Discussion

### 3.1. Microbial Characterization

Table 1 summarizes the results of the microbial analysis performed in meat products submitted to different smoking regimes. Total mesophilic bacteria counts reflect the higher counts obtained for LAB, as expected in dry-cured products, where these microorganisms are responsible for a slight fermentation during a long period [27,28]. The LAB counts reached 8.0 to 8.8 log cfu/g, which is in accordance with the values reported in dry-cured sausages by several authors [29,30,31]. *Staphylococci* counts were between 3.6 and 4.9 log cfu/g, with significantly lower counts in non-smoked control sausages. This fact reflects the higher LAB counts (8.8 log cfu/g) that outcompete *Staphylococci* in these non-smoked products, due to a higher acid production (lower pH values) and other factors related to the technological process [32,33,34]. Moreover, the dominant species in Portuguese dry-cured sausages is *Staphylococcus xylosus* [35], which is less tolerant to a decrease in pH, and the level of acidification is a key component of sausage fermentation and of microbiota modulation [33,36]. Furthermore, *Enterococci* were present among other LAB at a concentration between 3.15 and 4.21 log cfu/g, in agreement with the number of *Enterococci* present in other Mediterranean dry-cured sausages [29]. Enterobacteria counts were 2.1–2.7 log cfu/g without significant differences between the different smoking regimes. According to the UK guidelines for ready-to-eat foods, these values are borderline (2–4 log cfu/g) [37]. These results are similar to those reported before regarding dry-fermented sausages from Portugal and other Mediterranean countries [29,35]. However, these values are higher than those reported by others for Portuguese dry-fermented sausages [30,31], denoting the need to improve both the quality of the raw materials and the hygiene procedures.

### 3.2. Physicochemical Characterization and Sensory Analysis

The pH and a_W_ mean values were generally low, namely, 4.89–4.96 and 0.833–0.852, respectively, as it has been found in similar long dry-cured meat products [38,39]. Nevertheless, in non-smoked meat products, which had the highest level of LAB, the mean pH value was the lowest. The standardization of meat products weight losses was reflected by the similar a_W_ values obtained for all smoking regimes. The salt content of the studied dry-cured products ranged from 3.48% to 5.26%. The commercial formulation of the dry-cured sausages under analysis was not changed.

Table 2 shows the results obtained for color, textural parameters, and sensory attributes. A brighter (L*) and yellower (b*) color was observed for meat products submitted to longer smoking regimes (Table 2). The red (a*) color of the products was not affected by the smoking regime, since it is related to the nitrosomyoglobin pigment formed in cured meat products due to the addition of nitrite to the formula. Fat color was affected by the smoking regime, being more yellow and darker in products submitted to 60 h and continuous smoking regimes. Only slight changes in color were noticed, since color was measured in interior sliced sausages, and the deposition of smoking compounds occurs primarily on the surface of a product, with penetration increasing over time [40]. Therefore, the a* and b* values were slightly higher in sausages with longer smoking regimes, as has been reported by others for “chorizo” and Frankfurter-type sausages [40,41].

Regarding the texture profile analysis, significantly different adhesiveness, cohesiveness, and resilience values were observed according to the smoking regime (Table 2). It was noticed that lower adhesiveness and higher cohesiveness and resilience values were found in products with an effective smoking until reaching 38%–40% weight losses. Elias and co-workers [42] obtained similar texture values, although the products analyzed in the present study were harder, probably due to the lower a_W_ and pH values.

Concerning the sensory analysis (Table 2), the panel observed significant differences in color intensity, aroma intensity, hardness, fibrousness, succulence, salt perception, and overall appreciation. The meat products subjected to the longer smoking periods were considered to be harder by the panelists, which agrees with the opinion expressed by the sensory panel of the work described by Carrapiso et al. [43]. Salt perception was higher for sausages from the smoking regime B. Sausages subjected to the smoking regimes C and D were evaluated by the panelists to possess intense smoking flavor and aroma, which could influence salt perception. Regarding the non-smoked sausages, the panelists reported the samples to be raw and exhibit a characteristic casing flavor, as negative attributes. The overall appreciation was higher for meat products smoked for 20 h.

### 3.3. Polycyclic Aromatic Hydrocarbons

**Figure 1:**
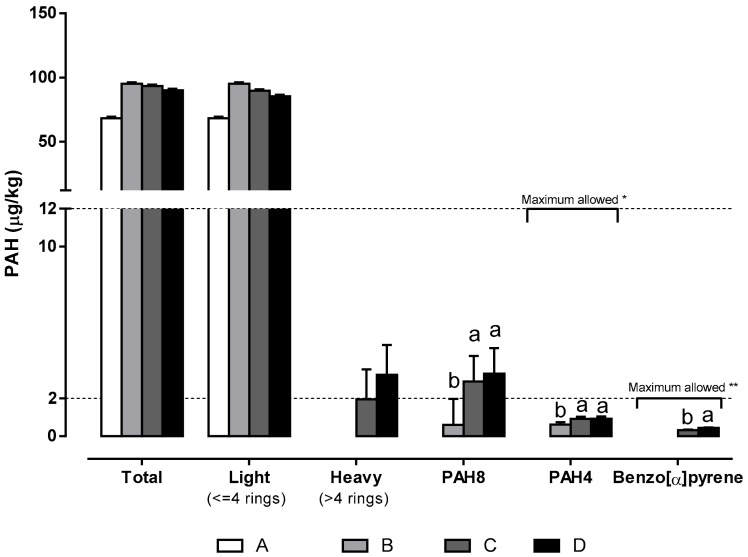
Polycyclic aromatic hydrocarbons (PAHs) (μg/kg wet weight) present in dry-cured sausages under different smoking regimes. Different letters for each group of PAHs represent significantly different means. * Maximum value of PAH4 allowed: 12 µg/kg; ** Maximum value of Benzo[α]pyrene allowed: 2 µg/kg. (**A**) no smoking; (**B**) 20 h effective smoking; (**C**) 60 h effective smoking; (**D**) effective smoking until reaching 38%–40% weight losses.

The total PAHs and light PAHs (<4 rings) mean concentration values (Figure 1) were generally low, when compared to those reported in other studies [26], and did not differ significantly in meat products submitted to the four different smoking regimes. Nevertheless, non-smoked sausages always showed lower PAHs values for all PAHs groups. Multiple factors could explain the PAHs content and profile in dry-cured meat products. The obtained results could be related to the fact that the deposition of PAHs occurs mainly on the surface of products, without massive diffusion to the inside [44]. *Paio* is a traditional large-caliber dry-cured meat product approximately 20–30 cm long. When compared to small-caliber meat products of a similar size, the mean content of PAHs (μg/kg product) could be lower, as mentioned before [26]. Moreover, all dry-cured meat products (smoked and non-smoked) were dried until they achieved the same 38%–40% weight losses and similar water activity values, thus minimizing any variability in the content of PAHs related to the drying/curing process.

Heavy PAHs were only detected in the products subjected to longer smoking periods (C and D). Eight PAHs (PAH8, benzo[α]pyrene, benz[α]anthracene, benzo[α]fluoranthene, benzo[k]fluoranthene, benzo[ghi]perylene, chrysene, dibenz[a,h]anthracene, and indeno[1,2,3-437 cd]pyrene) were present in all smoked meat products, with significantly higher values in those subjected to longer smoking periods (C and D). This same profile was observed for the four PAHs PAH4, benzo(α)pyrene, benz(α)anthracene, benzo(β)fluoranthene, and chrysene. Benzo(α)pyrene was detected only in products subjected to the smoking regimes C and D, with a significantly higher concentration in those obtained with the longer regime D. Nevertheless, the levels of all these hazardous PAHs were below the maximum limits established by the Commission Regulation (EC) No. 1881/2006 [45] in its consolidated version of 19 March 2018, namely, 12 μg/kg wet weight for PAH4 and 2 μg/kg wet weight for benzo(α)pyrene.

Table 3 shows the content of individual PAHs under different smoking regimes. The most abundant PAHs were naphthalene and phenanthrene. The contents of individual PAHs was generally similar to those previously determined for distinct Portuguese and Serbian fermented sausages [26]. A different screening of Portuguese traditional smoked meat products reported significantly higher PAHs values [46]. The observed difference is due to the distinct ways of expressing the results: our results were expressed in μg/kg wet weight according to the units patent in the legislation [45], while the values reported by Santos et al. [46] were expressed in μg/kg dry weight.

The processing of traditional dry-cured sausages of different types and in different countries usually includes a smoking step. It is essential to monitor the PAHs content in the final meat products, optimizing the smoking step to a maximum of 20 h cold smoking, evaluating raw materials contamination, and using other technological strategies to control and reduce the presence of potentially carcinogenic chemicals in dry-cured meat products [47].

Nevertheless, the presence of PAHs in dry-cured meat products is due not only to the PAHs released and deposited on the surface of sausages during the smoking process but also to spices and/or aromatic herbs used for seasoning [48,49].

## 4. Conclusions

Chemical safety of dry-cured meat products can be assured through the optimization of the smoking regime. In the present study, we have demonstrated that a reduced smoking step allows the control of PAHs levels in dry-cured meat sausages, while maintaining the products’ sensory characteristics. A good manufacturing process and an adequate selection of raw materials should be considered in order to avoid contamination.

Future perspectives include the study of low-salt dry-cured meat products in association with the control of the smoking step, in order to guarantee the stabilization of dry-cured meat products without increasing the content of PAHs.

## Figures and Tables

**Table 1:** Effect of smoking on microbiological and physiochemical parameters of dry-cured sausages.

Microbiological and Physicochemical Parameters	Smoking Regimes				*p*-Value
	A	B	C	D	
Mesophiles	8.01 ^a^ ± 0.22	7.27 ^b^ ± 0.09	7.34 ^ab^ ± 0.12	7.43 ^ab^ ± 0.27	*p* = 0.034 *
Lactic acid bacteria (LAB)	8.80 ^a^ ± 0.13	8.12 ^b^ ± 0.05	8.02 ^b^ ± 0.09	8.01 ^b^ ± 0.11	*p* = 0.000 ***
Staphylococci	3.59 ^b^ ± 0.29	4.57 ^ab^ ± 0.24	4.89 ^a^ ± 0.17	4.26 ^ab^ ± 0.34	*p* = 0.011 *
Enterococci	3.15 ± 0.18	4.21 ± 0.30	4.04 ± 0.39	3.89 ± 0.33	*p* = 0.094
Enterobacteria	2.25 ± 0.31	2.39 ± 0.62	2.71 ± 0.34	2.06 ± 0.36	*p* = 0.738
pH	4.89 ± 0.04	4.93 ± 0.05	4.95 ± 0.05	4.96 ± 0.04	*p* = 0.629
a_W_	0.852 ± 0.011	0.844 ± 0.009	0.833 ± 0.010	0.840 ± 0.010	*p* = 0.619
salt content (%)	4.37 ± 0.46	4.77 ± 0.15	4.55 ± 0.35	4.56 ± 0.27	*p* = 0.836

(A) no smoking; (B) 20 h effective smoking; (C) 60 h effective smoking; (D) effective smoking until reaching 38%–40% weight losses. Microbial counts are expressed in log colony-forming units (cfu)/g (mean ± SEM); a_W_: water activity. Within the same row, different letters (^a^ and ^b^) represent significantly different arithmetic means (Tukey’s honest significant difference (HSD) test). Significance: * *p* < 0.05, *** *p* < 0.001.

**Table 2:** Effect of smoking on color, textural parameters, and sensory attributes of dry-cured sausages.

Color, Textural Parameters, and Sensory Attributes	Smoking Regimes				*p*-Value
	A	B	C	D	
L*	38.48 ^bc^ ± 0.39	36.97 ^c^ ± 0.51	39.75 ^ab^ ± 0.44	40.36 ^a^ ± 0.47	*p* = 0.000 ***
a*	17.68 ± 0.39	16.81 ± 0.47	18.10 ± 0.37	18.05 ± 0.43	*p* = 0.107
b*	10.53 ^ab^ ± 0.49	9.19 ^b^ ± 0.46	10.42 ^ab^ ± 0.47	11.30 ^a^ ± 0.57	*p* = 0.029 *
Hardness (N)	58.82 ± 2.18	66.87 ± 1.94	64.70 ± 2.72	59.28 ± 3.08	*p* = 0.060
Adhesiveness (N × s)	−2.47 ^b^ ± 0.25	−3.28 ^a^ ± 0.24	−2.42 ^bc^ ± 0.22	−1.63 ^c^ ± 0.16	*p* = 0.000 ***
Cohesiveness	0.60 ^ab^ ± 0.01	0.58 ^b^ ± 0.01	0.59 ^ab^ ± 0.01	0.60 ^a^ ± 0.01	*p* = 0.020 *
Springiness	0.87 ± 0.01	0.87 ± 0.01	0.86 ± 0.01	0.91 ± 0.03	*p* = 0.293
Resilience	0.15 ^ab^ ± 0.00	0.13 ^b^ ± 0.00	0.14 ^ab^ ± 0.00	0.16 ^a^ ± 0.00	*p* = 0.001 **
Chewiness (N × mm)	30.97 ± 1.29	33.66 ± 1.23	33.03 ± 1.77	32.55 ± 2.09	*p* = 0.689
Color intensity	69 ^b^ ± 2	76 ^a^ ± 1	66 ^b^ ± 1	70 ^b^ ± 1	*p* = 0.000 ***
Aroma intensity	70 ^ab^ ± 2	73 ^a^ ± 3	65 ^b^ ± 2	65 ^ab^ ± 2	*p* = 0.017 *
Flavor intensity	68 ± 2	72 ± 1	67 ± 1	70 ± 2	*p* = 0.117
Hardness	52 ^b^ ± 1	51 ^b^ ± 1	53 ^ab^ ± 1	56 ^a^ ± 1	*p* = 0.019 *
Fibrousness	24 ± 3	20 ± 4	34 ± 3	35 ± 4	*p* = 0.019 *
Succulence	65 ^ab^ ± 2	68 ^a^ ± 2	63 ^ab^ ± 2	57 ^b^ ± 2	*p* = 0.010 *
Off colors	0 ± 0	0 ± 0	0 ± 0	0 ± 0	*p* = 0.470
Off aromas	2 ± 1	1 ± 0	1 ± 1	1 ± 0	*p* = 0.233
Off flavors	3 ± 1	1 ± 1	5 ± 1	3 ± 1	*p* = 0.074
Salt perception	51 ^c^ ± 1	60 ^a^ ± 2	54 ^bc^ ± 1	58 ^ab^ ± 1	*p* = 0.000 ***
Overall appreciation	66 ^ab^ ± 14	69 ^a^ ± 9	62 ^b^ ± 13	61 ^b^ ± 11	*p* = 0.019 *

(A) no smoking; (B) 20 h effective smoking; (C) 60 h effective smoking; (D) effective smoking until reaching 38%–40% weight losses. L*: light, a*: red, b*: yellow. Values are represented as mean ± SEM. Within the same row, different letters (^a,b^ and ^c^) represent significantly different arithmetic means (Tukey’s HSD test). Significance: * *p* < 0.05, ** *p* < 0.01, *** *p* < 0.001.

**Table 3:** Individual PAHs (µg/kg wet weight) determined in dry-cured sausages under different smoking regimes.

PAHs	Smoking Regimes				SEM	*p*-Value
	A	B	C	D		
naphthalene (NAP)	38.53	46.04	45.91	42.23	6.988	0.765
acenaphthylene (ACY)	2.77	4.73	6.90	6.47	1.418	0.208
acenaphthene (ACE)	1.37 ^ab^	1.97 ^a^	1.28 ^ab^	1.12 ^b^	1.194	0.013 *
fluorene (FLR)	3.68	5.67	6.41	3.87	1.260	0.199
phenanthrene (PHE)	10.57	24.97	14.67	17.25	6.616	0.104
anthracene (ANT)	0.53	1.56	2.07	2.11	1.536	0.050
fluoranthene (FLT)	1.79	2.49	2.44	3.01	1.164	0.140
pyrene (PYR)	1.82	2.40	1.95	2.81	1.169	0.219
benzo[a]anthracene (BaA)	n.d.	1.22 ^a^	0.39 ^b^	0.21 ^b^	0.179	0.002 **
chrysene (CHR)	n.d.	0.55	0.54	0.57	0.049	0.947
benzo[α]pyrene (BaP)	n.d.	n.d.	0.32 ^b^	0.44 ^a^	0.028	0.015 *
benzo[β] fluoranthene (BbF)	n.d.	n.d.	n.d.	n.d.	n.d.	-
benzo[k]fluoranthene (BkF)	n.d.	n.d.	n.d.	n.d.	n.d.	-
indeno[1,2,3-c,d]pyrene (IcP)	n.d.	n.d.	1.78	0.90	0.791	0.289
dibenzo[a,h]anthracene (DhA)	n.d.	n.d.	3.37	0.56	3.464	0.611
benzo[g,h,i]perylene (BgP)	n.d.	n.d.	0.93^b^	2.86 ^a^	0.438	0.009 **

(A) no smoking; (B) 20 h effective smoking; (C) 60 h effective smoking; (D) effective smoking until reaching 38%–40% weight losses. n.d.: not detected; the levels of individual PAHs are mean values. Within the same row, different letters (^a^ and ^b^) represent significantly different arithmetic means (Tukey’s HSD test). Significance: * *p* < 0.05, ** *p* < 0.01.
